# Conclusiveness, linguistic characteristics and readability of Cochrane plain language summaries of intervention reviews: a cross-sectional study

**DOI:** 10.1186/s12874-022-01721-7

**Published:** 2022-09-10

**Authors:** Aleksandra Banić, Mahir Fidahić, Jelena Šuto, Rea Roje, Ivana Vuka, Livia Puljak, Ivan Buljan

**Affiliations:** 1 Translational Research in Biomedicine, University of Split, School of Medicine, Šoltanska 2, Split, 21000 Croatia; 2 Faculty of Medicine, University of Tuzla, Dr. Tihomila Markovića 1, Tuzla, 75000 Bosnia and Herzegovina; 3 Department of Oncology, School of Medicine, University Hospital Center Split, University of Split, Spinčićeva 5, Split, 21000 Croatia; 4 Department of Research in Biomedicine and Health, University of Split, School of Medicine, Šoltanska 2, Split, 21000 Croatia; 5 Technology Transfer Office, Department for Science and Innovation, University of Split, Ruđera Boškovića 31, Split, 21000 Croatia; 6 Center for Evidence-Based Medicine and Healthcare, Catholic University of Croatia, Ilica 242, Zagreb, 10000 Croatia; 7 Department of Research in Biomedicine and Health, Cochrane Croatia, University of Split, School of Medicine, Šoltanska 2, Split, 21000 Croatia

**Keywords:** Cochrane plain language summary, Conclusiveness, Readability, LIWC (linguistic inquiry and word count)

## Abstract

**Background:**

One of the most important formats to disseminate the evidence in health to different populations are Cochrane Plain Language Summaries (PLSs). PLSs should be written in a simplified language, easily understandable and providing clear message for the consumer. The aim of this study was to examine the extent to which PLSs are customized for lay persons, specifically by providing conclusive, comprehensible, and readable messages.

**Methods:**

The study analyzed Cochrane PLSs of interventional studies (*N* = 4360) in the English language published from 1995 to 2019. We categorized the conclusiveness into one of the following categories: “positive”, “positive inconclusive”, “no evidence”, “no opinion”, “negative”, “negative inconclusive”, “unclear”, “equal”, “equal inconclusive”. Language characteristics were analyzed using Linguistic Inquiry and Word Count (LIWC) software. The level of readability was measured by SMOG (Simple Measure of Gobbledygook) index, indicating the number of years of education required to read the text. For each PLS, we also collected the following data: Cochrane Review Network, year of publication and number of authors.

**Results:**

Most of the PLSs (80%) did not have a conclusive message. In 53% PLSs there was no concluding opinion about the studied intervention or the conclusion was unclear. The most frequent conclusiveness category was “no opinion” (30%), and its frequency increased over time. The conclusiveness categories were similarly dispersed across Cochrane Networks. PLSs were written in an objective style, with high levels of analytical tone and clout above neutral, but a lower relation to authenticity and tone. The median number of years of non-specific education needed to read the PLSs was 14.9 (IQR 13.8 to 16.1), indicating that the person needs almost 15 years of general education to read the content with ease.

**Conclusion:**

Most of the Cochrane PLSs provided no concluding opinion or unclear conclusion regarding the effects of analyzed intervention. Analysis of readability indicated that they may be difficult to read for the lay population without medical education. Our results indicate that PLSs may not be so plain, and that the writing of Cochrane PLSs requires more effort. Tools used in this study could improve PLSs and make them better suited for lay audiences.

**Supplementary Information:**

The online version contains supplementary material available at 10.1186/s12874-022-01721-7.

## Background

It is important to ensure that the public and patients read information about health from reliable sources and that this information is adequate for the intended audience, providing the best evidence from research to help make informed choices about health [1, 2]. Acknowledging the purpose of communication to lay audiences, some medical journals, publishers, and networks provide free access to abstracts, summaries, or digests, written in plain language, either by the authors or the editors [3].

One of those networks is Cochrane, a global independent network that promotes evidence-informed health decision-making. It is an excellent source of high-quality and reliable information that is of interest to consumers. All Cochrane systematic reviews include plain language summaries (PLSs), short summaries written in simple language, freely available to the public on the web page of The Cochrane Library [4, 5]. Cochrane produces its reviews through eight thematic Networks of Cochrane Review Groups, each specified for a different area of health and medicine [6]. It is possible that different Cochrane entities have heterogeneous approaches to writing PLSs.

Following general guidelines for successful communication, in 2012, Cochrane started developing Standards for the reporting of PLSs in new Cochrane Intervention Reviews (PLEACS), with recommendations for writing PLSs, which should be understood by most readers without a university education [7]. However, analysis of 1738 Cochrane PLSs published from March 2013 to the end of January 2015 indicated that those PLSs had low adherence to the PLEACS standards, and they did not necessarily avoid complex language [8].

In 2019, members of Cochrane have also published additional guidance to supplement the PLEACS and help researchers to compose PLSs [9], and Cochrane continues with its projects to improve PLSs [10, 11]. This may be useful since special care needs to be taken when preparing evidence summaries for consumers, whereas effective communication must accommodate the audience’s capacity to read and understand the conveyed message [12].

Several recent studies have analyzed the content of Cochrane PLSs and their readability and linguistic characteristics [13, 14]. A study that assessed the readability of Cochrane summaries found that readers had to have a higher level of education to read PLS; however, the findings were limited to a small sample of PLSs as the study included 454 PLSs [15]. In addition to readability levels, linguistic characteristics, emotional tone, and perceived objectivity are also important aspects to consider when writing for consumers because it may affect the level of readers’ attention to the text [16].

Also, it would be useful if systematic reviews would provide clear conclusions and recommendations. Although there are studies available that investigated the conclusiveness of Cochrane reviews from specific fields [17, 18] to the best of our knowledge, there are no large-scale studies about the conclusiveness of Cochrane PLSs in the published literature.

Since all previous studies used small samples and were focused on only one aspect of conclusiveness, linguistic characteristics, or readability of PLSs, this study aimed to conduct a comprehensive analysis of those three aspects on all the available Cochrane PLSs of intervention studies published in English until February 2019. We also aimed to analyze trends of these variables in the analyzed time frame, and to explore whether these variables differed across different Cochrane Networks.

## Methods

### Study design, settings, and eligible summaries

In our cross-sectional study, we included all Cochrane PLSs of systematic reviews of intervention studies in the English language published until February 2019. For Cochrane reviews that had multiple versions, we analyzed the last English version of PLSs. The full texts of PLSs were extracted and made available to us by Cochrane. Only systematic reviews of interventions were eligible for inclusion.

### Outcomes

#### Conclusiveness

PLSs were analyzed for conclusiveness by assessing whether they contain conclusive recommendations (i.e., clear conclusions about whether an intervention is efficacious or not, and safe or not). Although the conclusiveness categories have been proposed by several previous studies [19–22], we chose a more structured categorization. We allocated summaries in one of the nine following categories [23]:

1. positive – there is (moderate/high quality) evidence of effectiveness/safety, i.e., the intervention was proven effective/safe;
2. positive inconclusive – there is evidence of effectiveness/safety, yet it is of a low quality/ inconclusive or authors state that more research is required);
3. no evidence – there is no evidence from RCTs because the literature search did not result in any eligible studies, i.e. empty reviews;
4. no opinion – the authors provided no opinion;
5. negative – there is (moderate/high quality) evidence of no effect or evidence of harm (ineffectiveness/harmful) or authors advised against the intervention/comparison, or it is not recommended;
6. negative inconclusive – there is evidence of ineffectiveness/harm (evidence show that there was no effect or the intervention was not safe) or authors advised against the intervention/comparison or it is not recommended; yet the evidence is of a low quality/inconclusive or authors state that more research is required;
7. unclear – more research is needed (authors state that more research is required);
8. equal – analyzed interventions were of equal effectiveness/safety;
9. equal inconclusive – interventions are equally effective/safe; yet the evidence is of a low quality/inconclusive, or authors state that more research is required.

Each PLS was allocated to a single conclusiveness category. Some of PLSs assessed multiple outcomes, and the decision to categorize PLS as positive, negative or equal, was based on the conclusions related to the primary outcome, not secondary outcomes. In cases of multiple primary outcomes, a discussion between assessors was made to place the PLS in conclusiveness category based on the overall recommendations of the PLS.

Conclusiveness was analyzed by two independent authors (AB, MF) who first performed a pilot study, with a sample of the first 100 PLSs. After reconciling the criteria between the two authors, it was sent to the other authors for review and agreement. This procedure enabled two independent authors (AB, MF) to harmonize criteria in the process of assessment of the conclusiveness and resolve possible subjectivity/cognitive bias accordingly.

#### Linguistic characteristics

We analyzed the PLSs for their linguistic characteristics with the software for language analyses (Linguistic Inquiry and Word Count – LIWC; http://liwc.wpengine.com/), resulting in summary variables: analytical thinking, clout, authenticity, emotional tone, and word count variable, which can be scored from 0 to 100. The analytical thinking variable is related to the style of writing. A higher score indicates that the text is recognized as more formally, logically, and hierarchically written. Clout score values are related to confidence and certainty, while a lower score means a more tentative tone of the text. Higher authenticity of a person indicates using more personal pronouns in the first person, singular forms, and verbs in present tense and relativity words. Emotional tone scores are higher when the text reflects more positive emotions [24].

#### Readability level

Each Cochrane PLS was analyzed using the quanteda.textstats package in R programming software (https://quanteda.io/), resulting in the level of education required for reading Cochrane PLSs expressed as SMOG (Simple Measure of Gobbledygook) index. SMOG index is expressed in numbers and represents the number of years of education needed to read the analyzed text without difficulties [25].

#### Systematic review information

For each PLS, we collected data about the corresponding Cochrane Review Network (Abdomen and Endocrine Network, Acute and Emergency Care, Cancer, Children and Families, Circulation and Breathing, Mental Health and Neuroscience, Musculoskeletal, Oral, Skin and Sensory and Public Health and Health Systems Network); a number of review authors and the year when the review was published.

### Data analysis

The data on conclusiveness was presented as frequencies and percentages, while other variables were presented as medians with corresponding 95% confidence intervals. SMOG index variability was presented as the interquartile range (IQR). LIWC variables: analytical thinking, clout, authenticity, and emotional tone were presented as scores converted to percentiles from 0 to 100, while the word count variable is simply given as the number of words in each PLS. Spearman’s rho correlation coefficient with 95% confidence interval was used as a measure for an association between the year when the PLS was published, the number of authors in a review, word count, LIWC variables, and SMOG index of PLSs. For comparing the linguistic characteristics before and after implementation of PLEACS, we applied logistic regression in which linguistic variables were entered as predictors and PLS publication year was dichotomized depending were they published before PLEACS (2013 or earlier) or after (2014 or later). All analyses were made using R software version 4.0.0. (R Core Team, 2020), and graphs were created using Microsoft Excel® (https://office.microsoft.com/excel).

## Results

Out of all available PLSs in the dataset (total *N* = 4537), we excluded 177 reviews as they were overviews (reviews synthesizing systematic reviews), methodological systematic reviews, systematic reviews of diagnostic procedures, and flexible systematic reviews. We included 4360 PLSs on which the analysis was performed. Majority of PLSs were from the Children and Families Network (Table 1). The distribution of the analyzed PLSs across years is given in the Supplement 1, Table S1.

**Table 1:** Comparison of linguistic variables and readability level across different conclusiveness categories

	Conclusiveness Md (95% CI)									P*
	Positive (*n* = 229)	Positive inconclusive (*n* = 768)	No evidence (*n* = 438)	No opinion (*n* = 1301)	Negative (*n* = 146)	Negative inconclusive (*n* = 308)	Unclear (*n* = 1012)	Equal (*n* = 50)	Equal inconclusive (*n* = 108)	
SMOG index	14.8 (14.4 to 14.9)	15.0 (14.8 to 15.1)	15.1 (14.8 to 15.2)	14.9 (14.7 to 15.0)	15.0 (14.8 to 15.4)	14.9 (14.6 to 15.2)	14.9 (14.7 to 14.9)	15.5 (15.0 to 15.7)	15.0 (14.5 to 15.5)	0.403
Analytic	95.4 (94.7 to 96.1)	95.2 (95.0 to 95.4)	94.4 (93.9 to 94.9)^b^	94.6 (94.5 to 95.1)	94.6 (93.5 to 95.4)	95.7 (95.2 to 96.1)	94.8 (94.5 to 95.2)	96.1 (93.4 to 96.9)	96.0 (95.5 to 96.7)	< 0.001
Clout	57.2 (54.4 to 59.6)^c^	55.7 (54.7 to 56.7) ^c^	55.7 (54.0 to 57.0) ^c^	53.5 (52.6 to 54.5) ^c^	50.0 (46.1 to 50.0)	53.4 (52.0 to 55.2) ^c^	54.2 (53.3 to 55.4) ^c^	48.1 (43.3 to 51.4)	49.7 (47.0 to 51.8)	< 0.001
Authentic	23.6 (21.5 to 25.9)	27.5 (25.8 to 28.9)	21.1 (19.0 to 22.6) ^d^	28.0 (26.6 to 29.1)	24.3 (19.8 to 29.2)	27.7 (25.6 to 29.0)	26.6 (25.2 to 27.8)	26.6 (20.5 to 33.6)	28.7 (25.5 to 33.8)	< 0.001
Tone	19.6 (14.5 to 25.8)	18.0 (15.7 to 20.6)	25.8 (17.1 to 25.8)^e^	17.1 (15.6 to 19.0)	16.7 (13.4 to 19.8)	15.6 (13.3 to 18.2)	18.1 (16.6 to 20.5)	15.6 (7.96 to 25.8)	17.9 (15.1 to 21.2)	0.004
Word count	260 (229 to 297)	365 (346 to 378)^a^	220 (204 to 232)	364 (349 to 375) ^a^	208 (186 to 230)	298 (278 to 312)	369 (352 to 379) ^a^	296 (218 to 336)	356 (326 to 399)^a^	< 0.001

*Md* Median, *CI* Confidence interval, *SMOG* Simple Measure of Gobbledygook

*Kruskal Wallis nonparametric test with Dunn post hoc comparison

^a^ Significantly different from “No evidence” and “Negative” and “Equal” categories

^b^ Significantly different from “Positive”, “Negative” and “Equal inconclusive” categories

^c^ Significantly different from “Negative”, “Equal” and “Equal inconclusive” categories

^d^ Significantly different from “Positive inconclusive”, “No opinion”, “Negative inconclusive”, “Unclear” and “Equal inconclusive”

^e^ Significantly different from “Negative” and “Negative inconclusive”

## Conclusiveness

**Fig. 1:**
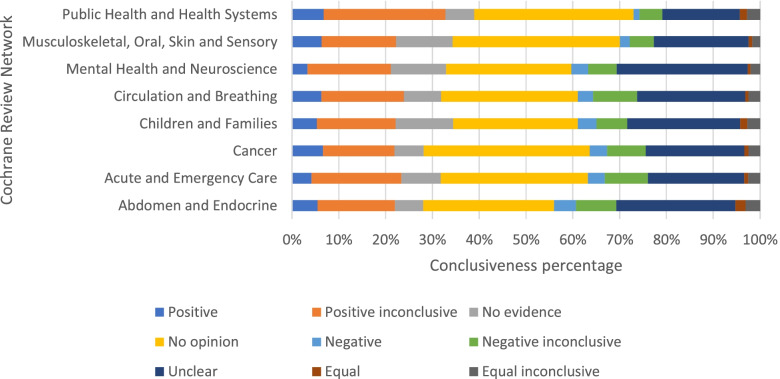
Distribution of conclusiveness categories across Cochrane Review Networks

**Fig. 2:**
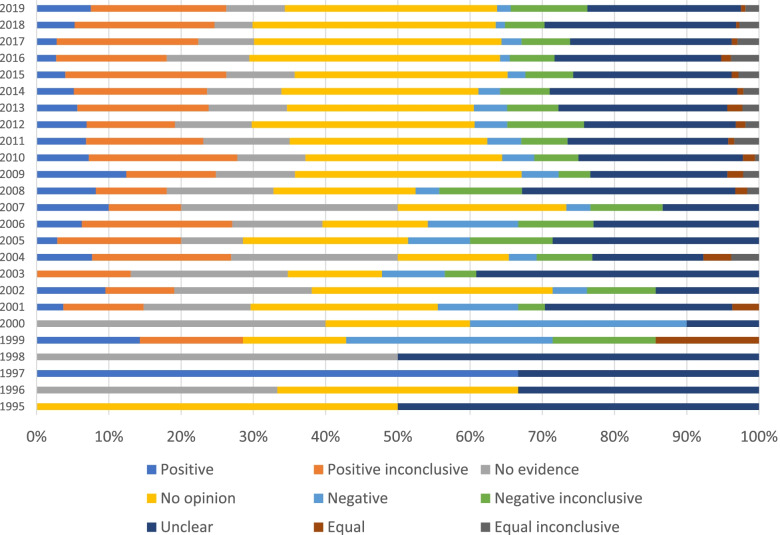
Distribution of PLSs by conclusiveness category across years

Most of the PLSs (80%) did not have a conclusive message for the readers. The most common conclusiveness category was the “no opinion” category (*N* = 1301; 30%), indicating that the PLS did not provide clear answers about the effectiveness of the therapy. The next most common category was the unclear conclusion (*N* = 1012; 23%). The differences in the frequency of different categories between Cochrane Networks were small (Fig. 1). As the number of PLSs increased over the years, the proportion of “vague” conclusions (“positive inconclusive”, “no evidence”, “no opinion”, “negative inconclusive”, “unclear”, “equal inconclusive”) remained stable (Fig. 2). Shortest PLSs were those categorized as negative conclusive and “no evidence” (Table 1).

### Linguistic characteristics

**Fig. 3:**
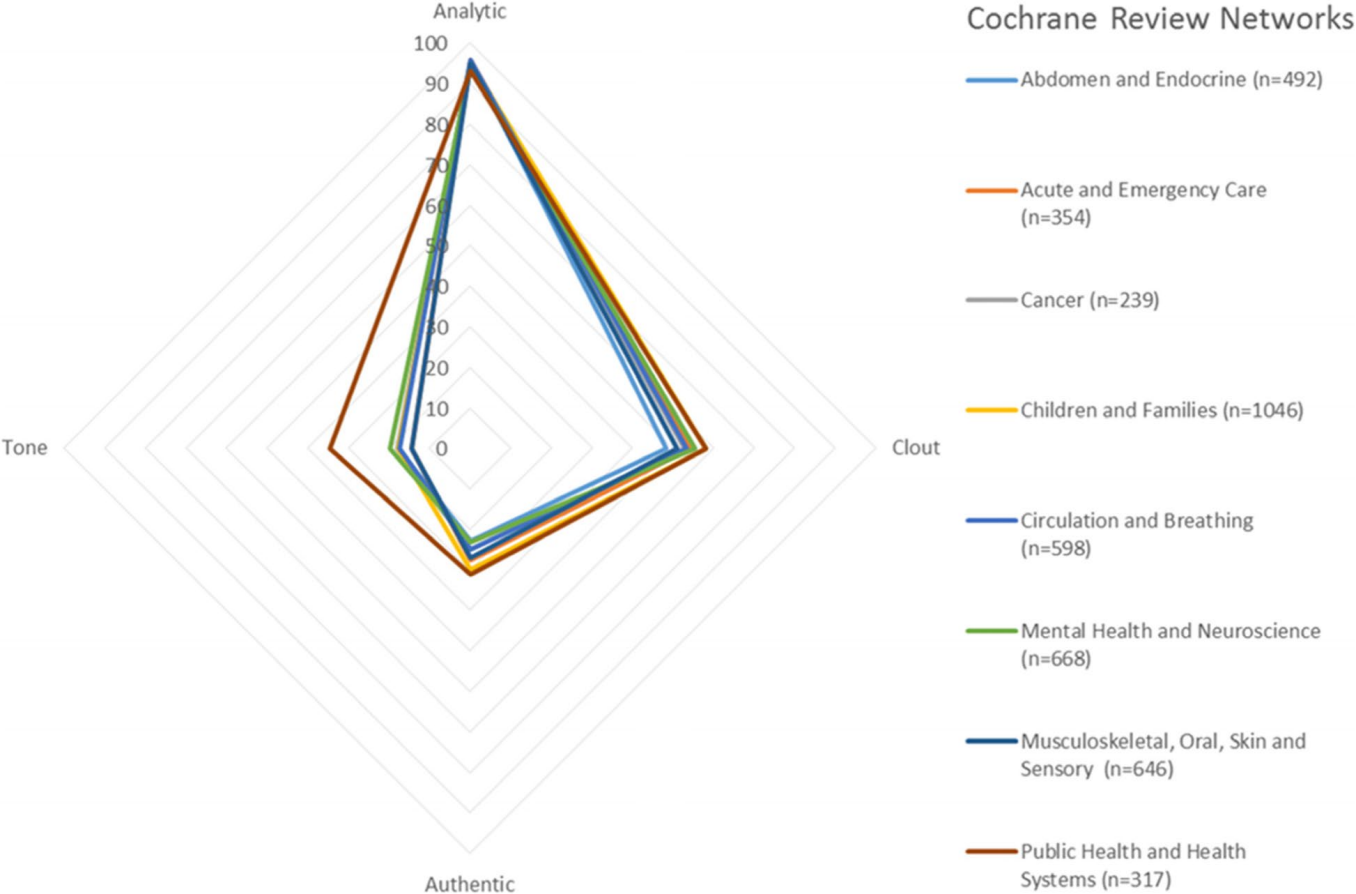
LIWC summary variables for Cochrane PLSs

LIWC analysis of summary variables showed a high percentage of text related to analytical writing, clout tendencies slightly above neutral, and a lower portion of text related to authenticity and tone (Table 1, Fig. 3).

The median number of words per summary was 332 (IQR 215–439), and the average number of words per single PLS increased in the observed period (Supplement 1, Table S2). On average, word count of PLSs was the highest in Cancer and Musculoskeletal, Oral, Skin and Sensory Network; lower, average numbers in PLSs were found in PLSs from Children and Families Network (Table 2).

**Table 2:** Comparison of linguistic variables and readability level across different Cochrane Review Networks

	Cochrane networks Md (95% CI)*								P*
	Abdomen and Endocrine (*n* = 492)	Acute and Emergency Care (*n* = 354)	Cancer (*n* = 239)	Children and Families (*n* = 1046)	Circulation and Breathing (*n* = 598)	Mental Health and Neuroscience (*n* = 668)	Musculoskeletal, Oral, Skin and Sensory (*n* = 646)	Public Health and Health Systems (*n* = 317)	
SMOG index	15.6 (15.5 to 15.8)	14.7 (14.4 to 14.9) ^a^	15.0 (14.7 to 15.4) ^a^	14.8 (14.6 to 14.9) ^a^	14.7 (14.5 to 14.8) ^a^	15.4 (15.2 to 15.5)	14.5 (14.3 to 14.4) ^a^	15.1 (14.8 to 15.2) ^a^	< 0.001
Analytic	95.8 (95.6 to 96.2) ^d^	95.7 (95.1 to 96.1) ^d^	94.9 (94.2 to 95.3)	95.1 (94.8 to 95.4) ^d^	95.7 (95.3 to 95.9) ^d^	94.0 (93.6 to 94.5)	94.8 (94.4 to 95.3)	93.2 (92.1 to 93.8)	< 0.001
Clout	48.4 (47.3 to 49.7) ^c^	54.2 (52.4 to 55.8) ^ac^	52.7 (50.0 to 54.2) ^ac^	58.1 (57.0 to 59.1) ^a^	53.3 (52.4 to 54.3) ^ac^	55.5 (54.4 to 56.4) ^ac^	51 (50.0 to 52.5) ^ac^	58.1 (56.6 to 59.9)^a^	< 0.001
Authentic	22.9 (20.8 to 24.4) ^c^	27.7 (25.8 to 29.7) ^a^	24.8 (22.2 to 27.2) ^c^	30.0 (28.7 to 31.8) ^a^	25.0 (24.0 to 26.4) ^c^	23.2 (21.6 to 24.8) ^c^	27.1 (25.2 to 28.3) ^ac^	31.1 (27.8 to 33.1) ^a^	< 0.001
Tone	17.7 (15.0 to 19.6) ^e^	14.4 (12.2 to 16.9) ^e^	17.9 (15.4 to 21.7) ^e^	17.7 (15.9 to 19.7) ^e^	17.4 (15.4 to 20.0) ^e^	19.7 (17.5 to 22.4) ^e^	14.5 (12.6 to 16.5) ^e^	34.6 (25.8 to 39.3) ^e^	< 0.001
Word count	313 (295 to 330) ^b^	366 (340 to 383) ^c^	392 (366 to 401) ^c^	294 (282 to 304) ^b^	308 (296 to 324) ^b^	322 (306 to 338) ^bc^	392 (378 to 406) ^bc^	344 (329 to 369) ^c^	< 0.001

*Md* Median, *CI* Confidence interval, *SMOG* Simple Measure of Gobbledygook

*Kruskal Wallis nonparametric test with Dunn post hoc comparison

^a^Significantly different from Abdomen and Endocrine Network and Cancer Network

^b^Significantly different from Cancer network and Musculoskeletal, Oral, Skin and Sensory Network

^c^ Significantly different from Children and Families network

^d^Significantly different from Mental Health and Neuroscience group and Public Health and Health Systems

^e^Significantly different than Public Health and Health Systems

### Readability level

Analysis of PLSs for the SMOG index showed that the median number of years of non-specific education needed to read the PLSs was 14.9 (IQR 13.8 to 16.1). Values of SMOG index slightly varied across Cochrane Review Networks. The PLSs from Abdomen and Endocrine Network had the highest average SMOG score and PLSs from Musculoskeletal, Oral, Skin and Sensory Network resulted in relatively the lowest SMOG score (Table 2).

### Temporal trends

Increasing number of authors, number of words, higher clout and authenticity tone, and a lower SMOG index, were the trends associated with the time flow, indicating that more recent PLSs were easier to read (Supplement 1, Table S2). The differences in linguistic characteristics of PLSs before and after implementation of PLEACS were small, although the it can be noted that they became slightly more readable after the implementation of PLEACS (Supplement 1, Table S3).

## Discussion

This study found that most PLSs (80%) did not end with conclusive messages about the studied interventions. Linguistic analysis of the PLSs found that the PLSs were not engaging enough for readers but written in mostly formal and “cold” style. The average readability level for Cochrane PLSs was slightly above recommended in terms of reading age and education degree for proper readability among lay audiences, indicating that PLSs may be difficult to read for persons without medical education.

Furthermore, recent PLSs were associated with a higher number of authors and words, higher clout and authenticity tone, and lower SMOG index, indicating they were easier to read. Our results indicate there are some improvements over the years that increase the readability of PLSs. However, further effort is needed to produce PLSs that will be more informative and more readable for the lay audience.

Over the years, the frequency of “non-conclusive” conclusions remained similar. More than half of the PLSs did not provide any opinion about the investigated intervention in its conclusions, or provided unclear conclusion, thus depriving the readers of the final message about the efficacy and safety of an intervention. The aim of Cochrane PLSs is to create health information that patients can understand and use [26]. Readers of PLSs likely seek a simple, clear and conclusive answer to their medical questions. It is acknowledged that systematic reviews were being criticized for not providing a specific guidance, and instead often concluding that there is little evidence to answer the question [27]. However, we need to distinguish PLSs that concluded there is “no evidence” from those that provided no opinion or unclear conclusion. It needs to be highlighted that lack of conclusiveness should not be considered a weakness of a systematic review or its PLS. If the PLS accurately reflects information from a systematic review, the PLS should not be judged as good or bad based on the conclusiveness. On the contrary, if a PLS does not contain a clear concluding message, or it does not have a final opinion at all about the studied intervention, this should be considered a poorly written PLS.

A higher proportion of non-conclusive results might also be due to a more reliable and critical approach to research practices and reporting. This can particularly relate to Cochrane reviews’ methodology as they are considered higher quality reviews [28]. Understanding inconclusiveness from the perspective of a lay person requires an awareness that methodologically sound systematic reviews are frequently inconclusive, a knowledge that comes with a certain level of science health literacy.

Comparing the results of the word count tests for inconclusive categories with the word count for conclusive categories of conclusiveness, it was visible that PLSs with vague conclusions were associated with a higher number of words in the review. Furthermore, PLSs categorized as “unclear” in terms of conclusiveness, resulted in the highest average word count score, implying that in cases with conflicting and debatable findings, summaries may require more words to support the explanation with broader, inconsistent reasoning, which led the authors to the inexplicit judgment. In general, as in the previous study [8], we found that Cochrane PLSs on average, were shorter than recommended by Cochrane [9], but we included additional variables and comparisons.

PLSs were found to have a relatively high number of words associated with analytical tone and clout, while levels of emotional tone were low, which is in line with results of the previous study on a smaller scale [15]. High levels of analytical tone suggest formal, logical, and hierarchical thinking [29], and these characteristics comply with the recommended form and structure of PLSs [7, 9]. Moreover, the authors of PLSs are mostly scientists, trained to think and write in a formal and logical style, and this style remained noticeable when they write for diverse readers. This phenomenon was also detected in the studies with students who were already trained to write formally. When they were asked to write in a less formal science style, their texts revealed a higher LIWC analytic score [30]. Therefore, higher analytical tone detected at PLSs is likely due to authors who are trained to write formal, and logical text structures.

Clout, a linguistic characteristic that implies confidence and expertise of the writer, was relatively high [19, 29]. Besides, higher clout found in PLSs complies with the consideration that the authors of PLSs are experts for the specific topic they write about. Lower numbers for authenticity in texts, found in PLSs, may be associated with a more guarded, distanced form of discourse [24]. Comparing the categories of conclusiveness, we found that authenticity was slightly higher in vague, inconclusive summaries. As higher authenticity suggests honest, personal and disclosing characteristics of the text [24], perhaps authors in summaries with higher authenticity did not want to overstate the efficacy of the described interventions, resulting without definite conclusions. Furthermore, “no evidence” conclusiveness category, which resulted in the lowest numbers for authenticity, was the one using the least proportion of first-person pronouns, since the authors of PLSs definitely declared in those cases that their search did not result in eligible studies or RCTs, and thus could not give personal recommendations.

In general, PLSs had low emotional tone levels as a summary variable of LIWC, which is associated with the negative emotional tone, such as anxiety, sadness, and hostility [24]. We assume that the reason of negative emotional tone lies in the presence of words related to negative emotions, such as pain, or disease. We noticed that texts from the Public Health and Health Systems (PH&HS) network provoke a slightly higher emotional tone in comparison to the other Cochrane networks. Unlike other networks, PLSs from the PH&HS network were associated with negative emotional tone to a lesser extent, probably due to its different thematic scope: from occupational health to global health, interventions related to consumers and communication [31], but still far from neutral, the middle level of emotional tone. PH&HS, together with the Children and Families network, also had the highest levels of clout and authenticity, making those two networks the most engaging ones compared to others. Future research should examine the reason for the existence of this difference, perhaps by using a text mining approach. We found that Cochrane networks differ in the number of words per review, with Cancer and Musculoskeletal, Oral, Skin and Sensory network having the highest number of words per review.

Most of the analyzed Cochrane PLSs were written with relatively high readability scores, which may not have an impact on comprehension of journalists, professionals, or audience with higher education. Yet, high readability could make PLSs difficult to read for the lay population without a university education, or a specific medical-oriented education or training. The readability score was similar across Cochrane Review Networks, as well as across different conclusion categories. In line with the studies which previously analyzed the readability of PLSs [32], we recommend PLS authors to use readability calculators as a tool in the process of writing PLSs. Acknowledging the considerable skills and time necessary to write a high quality PLS, authors may still simplify the language as much as possible. Difficulties with reading PLSs may prevent the public and patients from obtaining the best evidence from research, subsequently hindering the process of making informed decisions [33].

Our findings that showed an association between the year of publication and the number of authors are in line with the recent study that found an increasing number of authors in Cochrane reviews [34]. Moreover, our detection of an association between the number of authors and the number of words per PLS was consistent with results in other fields [35]. In addition to this, PLSs with higher word count showed slightly lower readability scores. This may lead to a possibility that contribution of each of the authors may result in additional text, resulting in longer texts, and longer texts allow explanations with higher number of simple, common, everyday words.

We also found that the PLSs written before the PLEACS standards were introduced differed in several dimensions compared to PLSs published after the PLEACS, although those differences were small. For this analysis we set the year 2014 as a cut-off since PLEACS were published in 2013 [7, 8]. After the introduction of PLEACS, the PLSs had lower readability scores and a slighty higher number of words related with analytic, authentic and emotional tone. However, those differences cannot be considered as causally associated with PLEACS, because we do not know if the writers of the PLSs were following PLEACS. A previous analysis on a large sample showed that PLSs rarely followed PLEACS [8]. Further guidance about writing PLSs, published in 2019 as a supplement for the PLEACS [9] was published after our search date, and thus could not influence our results.

Future guidance for writing PLSs should include advice for authors regarding writing clear conclusions and using tools that will improve linguistic characteristics and readability of those summaries. With the aim of resolving possible doubts or misunderstandings for lay readers who come across inconclusive PLSs, authors of these PLSs could refer to one of the categories we used for conclusiveness. Authors can explicitly declare that the specific systematic review may not provide a clear answer regarding anlyzed intervention, and recommend further engagement for lay reader. This engagement could consider following future research, or consulting medical specialists for personalized approach in cases where the consumer of PLS is a patient seeking for interventions for a specific health issue.

### Limitations

The findings of our study should be interpreted in view of several limitations. Conclusiveness was evaluated by two authors who independently read the reviews and made judgments about the category of conclusiveness for each review. These categorizations could be considered subjective; however, we did our best to use methods that are associated with minimization of bias. We used a pilot assessment, calibration exercise and consensus with the rest of the authors.

Our sample was large (*N* = 4360), as this study analyzed all PLSs published till February 2019, available to research team in early autumn 2019 when the analyses was initiated. However, it is acknowledged that it is possible that the PLSs published after February 2019 might have different characteristics. Therefore, the results of our study can not be generalized to the PLSs published outside of the time frame covered in this study.

SMOG readability formula was chosen among available readability formulas, as recommended and the best suited for health care applications [36]. Although a higher readability score indicates that the analyzed text could be difficult to read and consequently difficult to understand, we cannot automatically interpret texts with lower readability scores as more comprehensible/understandable to readers. It is acknowledged that the SMOG does not directly measure specific education needs in a deterministic way and does not take specific reader characteristics into account. Still, the assumption is that lower readability scores are a prerequisite for successful comprehension.

## Conclusions

Our study suggests that PLSs of Cochrane reviews could be improved in terms of phrasing of conclusions, linguistic characteristics, word count, and readability. While the linguistic characteristics of PLSs show an improvement over the analyzed years, the usability of PLSs for lay persons is likely decreasing as the number of PLSs with unclear conclusions is increasing. Tools used in this study can be employed by PLS writers to prepare summaries that will be better suited for the lay audience. Further studies should continue to assess the characteristics of new Cochrane PLSs periodically.

## Supplementary Information

**Additional file 1: Table S1.** Frequency distribution of the analyzed PLSs across years. **Table S2.** Correlation coefficients (Spearman rho correlation coefficient, 95% CI) between year when the plain language summary (PLS) was published, number of authors in a review, Word count, language characteristics and readability level of PLSs. **Table S3.** Logistic regression of prediction of plain language summaries (PLSs) written before the Standards for the reporting of PLSs in new Cochrane Intervention Reviews (PLEACS) were introduced; PLSs published before the year 2014 are labelled as 0 and those published in 2014 or after are labelled as 1.

## Data Availability

The data sets used and analyzed during the current study are publicly available on Open Science Framework website under this link: https://osf.io/qvu3a/.

## Abbreviations

PLS: Plain Language Summary
LIWC: Language Inquiry and Word Count
SMOG: Simple Measure of Gobbledygook readability formula
PLEACS: Plain Language Expectations for Authors of Cochrane Summaries
