# Supplementary material for: Conclusiveness, linguistic characteristics and readability of Cochrane plain language summaries of intervention reviews: a cross-sectional study
Source: BMC Med Res Methodol. 2022 Sep 10;22:240. doi: 10.1186/s12874-022-01721-7 (PMC9464378; doi:10.1186/s12874-022-01721-7)
Supplement: Supplementary file 1 — Additional file 1: Table S1. Frequency distribution of the analyzed PLSs across years. Table S2. Correlation coefficients (Spearman rho correlation coefficient, 95% CI) between year when the plain language summary (PLS) was published, number of authors in a review, Word count, language characteristics and readability level of PLSs. Table S3. Logistic regression of prediction of plain language summaries (PLSs) written before the Standards for the reporting of PLSs in new Cochrane Intervention Reviews (PLEACS) were introduced; PLSs published before the year 2014 are labelled as 0 and those published in 2014 or after are labelled as 1. [file 12874_2022_1721_MOESM1_ESM.docx]

**Supplement 1**

Table S1: Frequency distribution of the analyzed PLSs across years

| Year | Number of PLSs (n (%)) |
| --- | --- |
| 1995 | 2 (0.05) |
| 1996 | 3 (0.07) |
| 1997 | 3 (0.07) |
| 1998 | 2 (0.05) |
| 1999 | 7 (0.16) |
| 2000 | 10 (0.23) |
| 2001 | 27 (0.62) |
| 2002 | 21 (0.48) |
| 2003 | 23 (0.53) |
| 2004 | 26 (0.60) |
| 2005 | 35 (0.80) |
| 2006 | 48 (1.10) |
| 2007 | 30 (0.69) |
| 2008 | 61 (1.40) |
| 2009 | 137 (3.15) |
| 2010 | 180 (4.13) |
| 2011 | 234 (5.37) |
| 2012 | 376 (8.63) |
| 2013 | 479 (11.0) |
| 2014 | 466 (10.70) |
| 2015 | 529 (12.15) |
| 2016 | 516 (11.85) |
| 2017 | 505 (11.60) |
| 2018 | 475 (10.91) |
| 2019 | 160 (3.67) |

Table S2: Correlation coefficients (Spearman rho correlation coefficient, 95% CI) between year when the plain language summary (PLS) was published, number of authors in a review, Word count, language characteristics and readability level of PLSs

| Variable | Year | Number of authors | WC | analytic | clout | Authentic | Tone | SMOG |
| --- | --- | --- | --- | --- | --- | --- | --- | --- |
| Year |  |  |  |  |  |  |  |  |
| Number of authors | **0.22 (0.20 to 0.25)*** |  |  |  |  |  |  |  |
| WC | **0.58 (0.56 to 0.59)*** | **0.26 (0.23 to 0.29)*** |  |  |  |  |  |  |
| Analytic | -0.01 (-0.04 to 0.02) | -0.03 (-0.06 to 0.01) | 0.03 (0.00 to 0.06) |  |  |  |  |  |
| Clout | **0.20 (0.17 to 0.23)*** | **0.04 (0.01 to 0.07)*** | **0.11 (0.08 to 0.14)*** | -0.03 (-0.06 to 0.00) |  |  |  |  |
| Authentic | **0.15 (0.13 to 0.18)*** | 0.03 (-0.00 to 0.06) | **0.18 (0.15 to 0.21)*** | 0.01 (-0.02 to 0.04) | **-0.07 (-0.10 to -0.04)*** |  |  |  |
| Tone | -0.00 (-0.04 to 0.02) | -0.01 (-0.003 to 0.03) | **-0.07 (-0.10 to -0.04)*** | -0.05 (-0.08 to -0.02) | **0.08 (0.05 to 0.11)*** | -0.04 (-0.05 to 0.01) |  |  |
| SMOG index | **-0.15 (-0.18 to -0.12)*** | **0.06 (0.03 to 0.09)*** | **-0.07 (-0.10 to-0.04)*** | **0.22 (0.20 to 0.25)*** | **-0.13 (-0.16 to -0.10)*** | **-0.06 (-0.09 to -0.03)*** | **0.04 (0.01 to 0.07)*** |  |

*Statistically significant at P<0.05 level.

Table S3. Logistic regression of prediction of plain language summaries (PLSs) written before the Standards for the reporting of PLSs in new Cochrane Intervention Reviews (PLEACS) were introduced; PLSs published before the year 2014 are labelled as 0 and those published in 2014 or after are labelled as 1

| Variable | Odds ratio | 95% CI | P | McFadden R^2^ |
| --- | --- | --- | --- | --- |
| Intercept | 0.049 | 0.01 to 0.20 | <0.001 | 0.27 |
| SMOG | 0.88 | 0.84 to 0.92 | <0.001 |  |
| Analytic | 1.01 | 0.99 to 1.02 | 0.248 |  |
| Authentic | 1.01 | 1.01 to 1.01 | <0.001 |  |
| Clout | 1.03 | 1.02 to 1.03 | <0.001 |  |
| Tone | 0.99 | 0.99 to 1.00 | 0.019 |  |
| Word count | 1.01 | 1.01 to 1.01 | <0.001 |  |

SMOG-Simple Measure of Gobbledygook; CI- confidence interval.

Abbreviations:

CI- confidence interval

PLS – Plain Language Summary

WC – word count

LIWC – Language Inquiry and Word Count

SMOG – Simple Measure of Gobbledygook readability formula

PLEACS – Plain Language Expectations for Authors of Cochrane Summaries
